# Examination of the variables related to parents' irrational beliefs: a meta-analysis study

**DOI:** 10.3389/fpsyg.2025.1478262

**Published:** 2025-05-01

**Authors:** Ali Çekiç, Kübra Korkmaz, Sebnem Aslan Cömert, Ibrahim Yildirim

**Affiliations:** ^1^Department of Psychological Counseling and Guidance, Gaziantep University, Gaziantep, Türkiye; ^2^Izmir Provincial Directorate of National Education, Izmir, Türkiye; ^3^Gaziantep Provincial Directorate of National Education, Gaziantep, Türkiye; ^4^Department of Measurement and Evaluation in Education, Gaziantep University, Gaziantep, Türkiye

**Keywords:** parenthood, parenting beliefs, cultural differences, irrational beliefs, meta-analysis

## Abstract

**Introduction:**

The aim of this study was to determine the variables that have been studied in relation to parents' irrational beliefs, and to examine the relationships between these variables through meta-analysis. Additionally, this study aimed to explore cultural differences in parents' irrational beliefs by conducting subgroup analyses based on the country in which the study was conducted.

**Methods:**

Fifteen studies, which had been issued in the databases of Google Scholar and Web of Science (WOS) between the years of 1990 and 2023, met the inclusion criteria and were included in the analyses. In these fifteen studies, the associations of parents' irrational beliefs with twenty three different variables were examined. These variables were reorganized under the titles of “Irrational thoughts”, “Positive mental health (Parent)”, “Negative mental health (Parent)”, “Negative mental health (Child)”, “Positive behavior (Parent)”, “Negative behavior (Parent)”, “Adaptation (Parent)”, “Parent-child relationship”, “Positive characteristics (Child)” and “Irrational beliefs (Child)” in line with expert opinions. Among these variables, the effect sizes of those that included a sufficient number of studies for meta-analysis were calculated separately.

**Findings and conclusion:**

Accordingly, the largest effect size was calculated between parental irrational beliefs and general irrational beliefs (0.60), and the smallest effect size was calculated between parental irrational beliefs and children's negative mental health characteristics (0.15). In the subgroup analyses conducted according to the country of the study, the largest effect sizes were observed in USA for all variables, while the lowest effect sizes were observed in Turkey.

## 1 Introduction

According to Rational Emotive Behavior Therapy (REBT), which is put forward by Albert Ellis, events, cognitions, and outcomes are interrelated. According to this view, which is called the A-B-C model, obligations that include irrational evaluations can affect human behavior and lead to deterioration of mental health (Sharf, 2011; Terjesen and Kurasaki, 2009). REBT, which is also accepted as a psychotherapy model by clinicians (David et al., 2018), claims that people perceptions of the world are based on rational and irrational beliefs (Çivitçi, 2014; Türkçapar, 2020). According to Ellis, the aim of REBT-based practices is to make clients feel good about themselves by providing emotional and behavioral change. According to REBT, in order for this to happen, there must be fundamental changes in the mindsets that form their perspectives on current and future situations. This new perspective, which is intended to be given to the clients in the REBT process, should include some basic features such as tolerance to self and others, acceptance of uncertainty, flexibility, scientific thinking (Ellis, 1980). As the number of studies confirming the basic assumptions of REBT increases (Vîslǎ et al., 2016), the number of scale development studies to measure individuals' irrational beliefs in different roles including parenting (Gavita, 2011; Kaya and Hamamci, 2011), teaching (Gündüz, 2006) and in different areas of responsibility such as romantic relationships (Sari and Korkut Owen, 2015) and career choice (Erdem and ve Bilge, 2008) has also increased.

The increasing number of instruments developed to measure irrational beliefs about parenting also increases the number of studies on this subject. In the literature review conducted within the scope of the present research, seven different measurement tools used to measure parents' irrational beliefs were found (Ackerman, 1991; Campis et al., 1986; Gavita, 2011; Joyce, 1995; Kaya and Hamamci, 2011; Lieber et al., 2006; Roehling and Robin, 1986). Some of these measurement tools have been adapted to different cultures and used for research on parents from different cultural backgrounds.

According to REBT, parents' irrational beliefs may lead children to be affected by negative outcomes (Hocaoglu, 2016). It is expected that when parents acquire rational beliefs instead of irrational beliefs, their parenting behaviors will be more functional (Terjesen and Kurasaki, 2009). In fact, most of the initial emotions and cognitions regarding parenting are positive (Çekiç et al., 2019). However, parents also experience negative emotions and behaviors within the process. It is considered that these negative emotions and behaviors of parents may be due to their specific beliefs regarding parenting rather than their general irrational beliefs (Ellis et al., 1966; Joyce, 1989, 2006).

In the literature, there are some classifications concerning parents' beliefs. The most commonly used classification was made by Joyce (2006). Joyce (2006) examined the core irrational beliefs of parents and classified them into four groups. Demandingness: Parents' rigid beliefs that involve the statements of “should.” Catastrophizing: Parents' interpreting of parenting events in a much worse way than they really are. Low frustration threshold: Parents' beliefs that some events are unbearable. Appraisal of the individual's worth: In this type of belief, parents believe that an individual's worth consists of their skills and achievements, and can only be measured by these. According to Ackerman (1991), parents have two core beliefs about their parenting roles: irrational and rigid thinking. These beliefs consist of parents' unrealistic expectations about their children and themselves regarding their role as parents. In another study on the subject, Kaya and Hamamci (2011) classified irrational beliefs about parenting into two subgroups: expectations and perfectionism. Expectations describe parents' expectations about their relationships with their children, while perfectionism includes parents' perfectionist thoughts about raising children. It has been observed that parents, especially those who set high standards for their children, have high expectations from them, and find their children's efforts insufficient, are concerned about their children's achieving certain goals (Baytemir, 2019). Parents have concerns not only about their expectations from their children but also about their own competencies.

For example, high expectations concerning parenthood threaten especially the mental health of mothers, negatively affecting their life satisfaction and leading to some pathologies such as depression (Choi et al., 2007). Ellis and Harper (2005) state that parents' irrational beliefs not only lead to negative consequences such as anxiety, anger, depression, and worthlessness in themselves, but also lead to the formation of some unhealthy thoughts in their children. In addition, parents' irrational evaluations of themselves and their children can lead to problematic relationships between parents and children (Hortaçsu, 2012).

Measurement tools developed in different countries and revealing different classifications regarding parenting are important in terms of showing that elements of parenting can be affected by culture. As a matter of fact, according to Selin (2002), parents' parenting behaviors in different areas are influenced by the culture in which they live. For example, independent socialization skills for German middle class families, interdependent socialization skills for Cameroonian families, and autonomous relational skills for Costa Rican families are prioritized in child rearing (Keller et al., 2005). According to Harkness and Super (2002), in order to better evaluate the individual behaviors of parents in different cultural contexts, it is necessary to obtain more detailed and systematic data on beliefs, practices, and developmental processes of children. Our study aims to reveal the relationships between parenting beliefs, which are an important determinant of parenting behaviors, and both the characteristics of parents and the characteristics of children.

In line with the general purpose of the study, the first aim of this study is to determine the variables that are examined together with parents' irrational beliefs in correlational research. Another aim of the study is to examine the relationships between these variables through meta-analysis. Thus, more definitive findings regarding parental irrational beliefs will be presented to the researchers by determining the effect sizes related to the correlations between the variables. In addition, subgroup analyses will be conducted depending on the country in which the research was conducted to reveal cultural differences regarding parental irrational beliefs. The results to be determined through subgroup analyses may also contribute to a better understanding of cultural differences in irrational beliefs regarding parenting. For this purpose, answers to the following research questions were sought:

1. What are the variables related to parental irrational beliefs?
2. What are the mean effect sizes of the variables “irrational beliefs (parent),” “positive mental health (parent),” “negative mental health (child),” “positive behavior (parent),” and “negative behavior (parent)” whose correlation values were examined with parenting irrational beliefs?
3. Do the effect sizes obtained from the variables related to parental irrational beliefs differ depending on the country where the research conducted?

## 2 Method

Nowadays, listing the results of independent studies conducted on the same subject is one of the commonly used methods in many disciplines. The researchers state that the findings of the analyses obtained by collecting data from different studies on the same subject have higher statistical power than the findings based on a single study. For this reason, the accumulation of knowledge obtained from the results of many studies is considered as the cornerstone of science (Çarkungöz and Ediz, 2009). Meta-analysis is a quantitative method used to summarize findings by combining the results of multiple studies. Meta-analysis is used to obtain an overall result or summary results by quantitatively summarizing the results of multiple primary studies and includes a range of statistical methods (Arthur et al., 2001).

The present study aimed to reveal the variables associated with parents' irrational beliefs, and examine the relationships between these variables by meta-analysis. Thus, it was intended to reach a more comprehensive result by compiling the data obtained from the research on the subject of the study. PRISMA Guidelines (Page et al., 2021) was taken as basis in the meta-analysis process.

### 2.1 Data sources and search strategy

**Figure 1:**
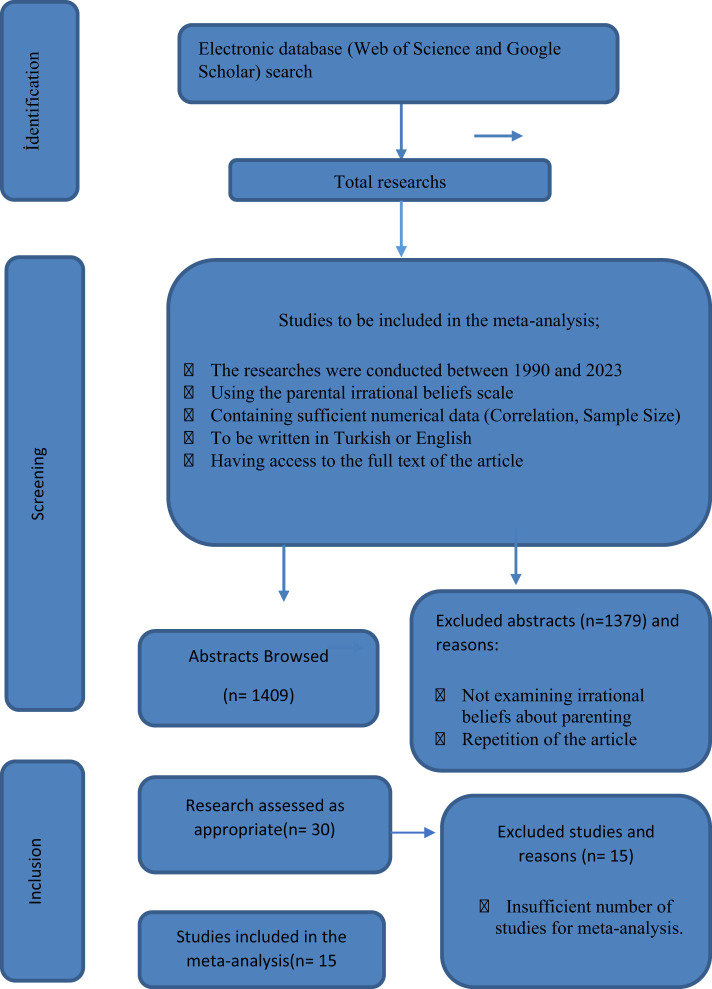
Flow chart.

The aim of the present study is to identify the variables associated with irrational beliefs about parenting through review and to examine these relationships. The searching process was completed in March 2023. Google Scholar and Web of Science (WOS) databases were searched from 1980 to 2023. For Google Scholar search, the pattern *allintitle: (“irrational beliefs” OR “beliefs”) AND (“parent” OR “parenting” OR “mother” OR “father”)* was used, and for WOS search, the pattern *Citation Report: Parent OR Parenting OR Farther OR Mother (Title) AND Belief OR Irrational Belief (Title) and 1980–2023 (Publication Years)* was used. The flow chart in which the procedures carried out in the research process are explained in detail is shown in Figure 1.

### 2.2 Inclusion criteria for analysis

All studies reported in the present meta-analysis include statistical data that are required to be used to calculate the effect size and allow us to examine the role of parents' irrational beliefs on various variables. Publication bias as stated by Rosenthal (1979) is a crucial threat to the validity of meta-analysis studies. In this context, all the scientific studies found in the researching process were included in the scope.

The studies concerning parents' irrational beliefs were first searched without setting a specific year limit. According to the present review, the earliest studies concerning parents' irrational beliefs were conducted by Ackerman (1991) and Starko (1991). For this reason, the studies included in the meta-analysis were published between the years of 1990 and 2023.

According to the review of the databases, the number of studies that addressed irrational beliefs regarding parenting and whose full text could be accessed was 30. The 30 studies on parents' irrational beliefs were re-evaluated for suitability for meta-analysis. One of these studies was excluded because it was not written in English or Turkish, five of them did not report correlation coefficients, and the remaining nine articles were excluded because they did not use a measurement tool developed to examine parents' irrational beliefs. As a result of these evaluations, 15 studies met the inclusion criteria. In these 15 studies, the correlations between parents' irrational beliefs and 23 different variables were examined (Table 1).

**Table 1:** Variables analyzed in relation to parents' irrational beliefs.

1	General irrational thoughts (parent)	13	Inconsistent parental discipline
2	Self-acceptance (parent)	14	Corporal punishment (parent)
3	Anxiety (parent)	15	Parental competence
4	Depression (parent)	16	Control focused parental attitude
5	Parental stress	17	Psychological violence (parent)
6	Negative parental behaviors	18	Adaptation skills (child)
7	Positive parental behaviors	19	Problem solving skills (child)
8	Couple compatibility (parent)	20	Irrational beliefs (child)
9	Parent-child relationship	21	Depression (child)
10	Caring for the child	22	Anxiety (child)
11	Neglectful parenting	23	Child's behavioral problems
12	Life satisfaction (parent)		

These variables were re-examined and some of the variables that were thought to represent a common theme were combined and grouped under a single heading. In Table 1, anxiety, depression, and stress variables reflecting the negative characteristics of parents' mental health were grouped under “Parent Negative Mental Health;” depression, anxiety, and behavioral problems of children were grouped under “Child Negative Mental Health;” positive parental behaviors, caring for the child and parental perception of competence were grouped under “Positive Parental Behaviors,” the variables of negative parental behaviors, neglectful parenting, inconsistent discipline, corporal punishment and psychological violence were combined under the heading of “Negative Parental Behaviors,” self-acceptance and life satisfaction were combined under the heading of “Parental Positive Mental Health” and finally child adaptation skills and child problem solving skills were combined under the heading of “Child Positive Characteristics” based on expert opinions (Table 2). The evaluations were conducted by two full-time faculty members who have studied on irrational beliefs of parents in the Department of Guidance and Psychological Counseling at Gaziantep University.

**Table 2:** Variables related to parents' irrational beliefs (integrated).

**References**	***n***	**Irrational thoughts (P)**	**Positive mental health (P)**	**Negative mental health (P)**	**Negative mental health (C)**	**Positive behav. (P)**	**Negative behav. (P)**	**Adaptation (P)**	**Parent-child relationship**	**Positive characteristics (C)**	**Irrational beliefs (C)**
McDonald, 1993	124			0.43				−0.26			
Roehling and Robin, 1986	60								0.63		
Starko, 1991	46			0.52							
Ackerman, 1991	129	0.69		−0.47		0.11	0.10				
Kaya, 2010	884	0.54		0.19							
Salhany, 2010	152			0.21	0.10	−0.36	0.60				
Gavita, 2011	212	0.54	−0.60								
Winters, 2012	44			0.55		−0.40	−0.35				
Kaya and Hamamci, 2013	489	0.36									
Çekiç et al., 2017	237				0.27				0.41		
Çekiç et al., 2019	318		−0.02								
Buga et al. (2019)	551					−0.04	−0.16				
Warren, 2021	101						0.50				
Koç-Arik (2021)	374				0.12	−0.03	0.00			0.07	
Uzun and Avcı (2021)	608				0.12			0.05		0.03	−0.01

(P), Parental characteristics; (C), Child's characteristics.

As a result of the evaluations, 10 different variables related to parental irrational beliefs were determined. To obtain sufficient variation between variables, analyses were not conducted for studies with two or fewer variables.

Different measurement tools assessing parents' irrational beliefs were found. Some of these scales included more than one subscale. If these instruments had a total score, their total scores were used. If there was not a total score, the correlation values related to the subscale that was considered to best reflect irrational beliefs regarding parenting were taken into consideration. For this purpose, the opinions of three different experts studying irrational beliefs in parenting were obtained. According to experts' opinions, the perfectionism subscale of the PIBS scale developed by the Parental Responsibility Subscale of the Parental Locus of Control Questionnaire (PLOC) developed by Campis et al. (1986), the Shaming subscale of the Chinese Child-Rearing Beliefs Questionnaire (CCRBQ), and the Irrational Beliefs subscale of the Parent Rational and Irrational Beliefs scale developed by Gavita et al. (2011) were used to measure parents' irrational beliefs. PIBS (Kaya and Hamamci, 2011) was developed with 520 parents and the Cronbach Alpha reliability coefficient of the scale was calculated as 0.80. PLOC (Campis et al., 1986) was developed with 147 parents and Cronbach Alpha reliability coefficient was calculated as 0.67. P-RIBS (Gavita et al., 2011) was developed with 287 parents aged 25–52 years and Cronbach alpha reliability coefficient was calculated as 0.42. CCRBQ was developed with 504 parents and Cronbach alpha reliability coefficient was calculated as 0.66.

### 2.3 Inter-rater reliability

Two authors of the present study performed coding in accordance with the coding procedure. The coding data obtained from the two authors were used to evaluate the reliability of the results of the present study. The correlation coefficient between the results determined by the two coders was calculated. The similarity value between the two raters was found as 1.0 (Sen and Yildirim, 2019).

### 2.4 Statistical analysis

Since the studies were obtained from different sources, the researchers used the random effects model. In cases that the random effects model did not provide statistically significant results the fixed effects model was preferred.

In the present meta-analysis, the relationships between two variables were discussed. The researchers used Fisher *z*-value to transform coefficients of correlation to obtain the results. The following formula is used to convert Pearson correlation coefficient (r) into Fisher *z* (Sen and Yildirim, 2020):


$$\begin{array}{l} z=0.5\times \ln\left(\frac{1+r}{1-r}\right) \end{array}$$

The variance of the z value is used as $V_{z}=\frac{1}{n-3}$. Thus, the standard error of the Fisher *z* value is calculated by the formula $S.E._{z}=\;\sqrt{V_{z}}$.

In order to assess the heterogenity, the publications included in the meta-analysis were initially examined with the *Q* statistic (Huedo-Medina et al., 2006). *I*^2^ values were also examined to obtain more comprehensive information regarding heterogeneity. Statistically significant *Q* statistics indicate heterogeneity. In addition, the calculated *I*^2^ value shows the degree of heterogeneity from 0% to 100%. The *I*^2^ value is calculated from the *Q* statistics and k in the formula indicates the number of studies included in the meta-analysis.


$$\begin{array}{l} I^{2}=\left(\frac{Q-k-1}{Q}\right).100 \end{array}$$

*I*^2^ values of 50% and 75% indicate moderate and high level of heterogeneity, respectively (Higgins and Thompson, 2002). In this study, cultural analyses were conducted to determine the source of heterogeneity. Because the studies included in the meta-analysis belong to studies conducted in different cultures (countries).

In the Parental Irrational Beliefs Scale (PIBS) developed by Ackerman (1991), unlike other parenting scales, a low score is regarded as having more irrational beliefs and a high score is regarded as having fewer irrational beliefs. Therefore, the negative correlations between PIBS and the variables were evaluated as positive considering the direction of other parental beliefs.

### 2.5 Investigation of publication bias

A researcher conducting a meta-analysis study is required to aim at accessing all relevant studies after identifying the research question. It may not be possible for the researcher to reach unpublished studies on a subject. Only published studies that have been reported significant results must be included in the meta-analysis (Greenhouse and Iyengar, 1994). Publication bias is a term used to indicate that statistically significant results are more likely to be published than non-significant results (Petitti, 2000). Moreover, when the published studies are examined, it is determined that statistically significant differences are more often found, and this situation causes publication bias in meta-analysis. The fact that the studies are mostly published in English or that there are many publications obtained from the same study also jeopardizes the validity of meta-analysis by leading to bias (Card, 2015). In this study, trim and fill, fail safe N and funnel plot methods were used to determine whether there was publication bias.

## 3 Results

In this section, the overall effect sizes obtained because of the meta-analysis of the variables gathered under five different headings related to parental irrational beliefs, the validity of the calculated effect size and subgroup analyses are presented under separate headings, respectively.

### 3.1 Examining the relationships between parents' irrational beliefs and general irrational beliefs

When the related keywords were searched, four different studies, in which investigating the relationships between parents' irrational beliefs regarding their parenting roles and the general irrational beliefs of Cognitive Behavioral Theories, were found. Under this title, the findings of the meta-analysis of the correlation coefficients obtained from the studies are presented. First, the overall effect size for the correlation coefficients obtained from four different studies was calculated and shown in Table 3.

**Table 3:** The results of the over all effect size about general irrational beliefs.

**Model**	***k***	**Fisher *z***	***z***	***SE***	***p***	**%95 CI**		***df***	***Q***	***p***	***I* ^2^**
						**Lower limit**	**Upper limit**				
Fixed effect	4	0.557	22.992	0.024	0.000	0.510	0.605	3	28.857	0.00	89.604
Random effects	4	0.597	7.150	0.083	0.000	0.443	0.760				

The effect size of the data of the studies included in the meta-analysis was estimated as 0.56 with 95% confidence intervals (0.51 and 0.61), based on the fixed effect model. According to the random effects model, it was estimated as 0.60 with 95% confidence intervals (0.44 and 0.76.). The calculated effect sizes indicate a large effect (Evans, 2023). The *Q* (*df* = 3) statistic for this analysis was calculated as 28.86 (*p* < 0.01). The estimated *I*^2^ is 89.60%, indicating the heterogeneity of the studies included in the analysis.

**Figure 2:**
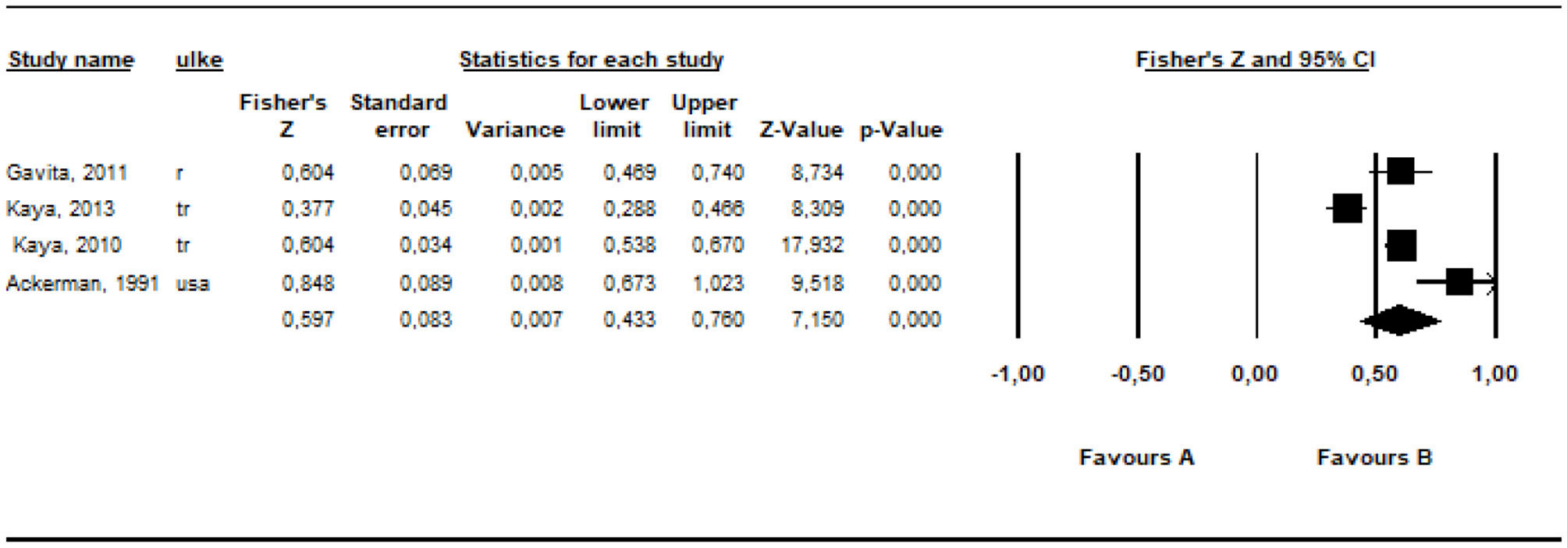
Forest plot showing the distribution of the effect size of the studies.

The *r* equivalent of the calculated Fisher *z* value of 0.597 was calculated as 0.535. This value is considered as a large effect for meta-analysis (Cohen, 1988). The forest plot showing the distribution of effect sizes according to the random effects model is presented in Figure 2.

According to the forest plot presented in Figure 2, the highest effect size was 0.85 by Ackerman (1991) and the lowest one was 0.38 by Kaya and Hamamci (2013). All of the calculated effect sizes are positive.

#### 3.1.1 The results of the validity of effect size estimates

**Figure 3:**
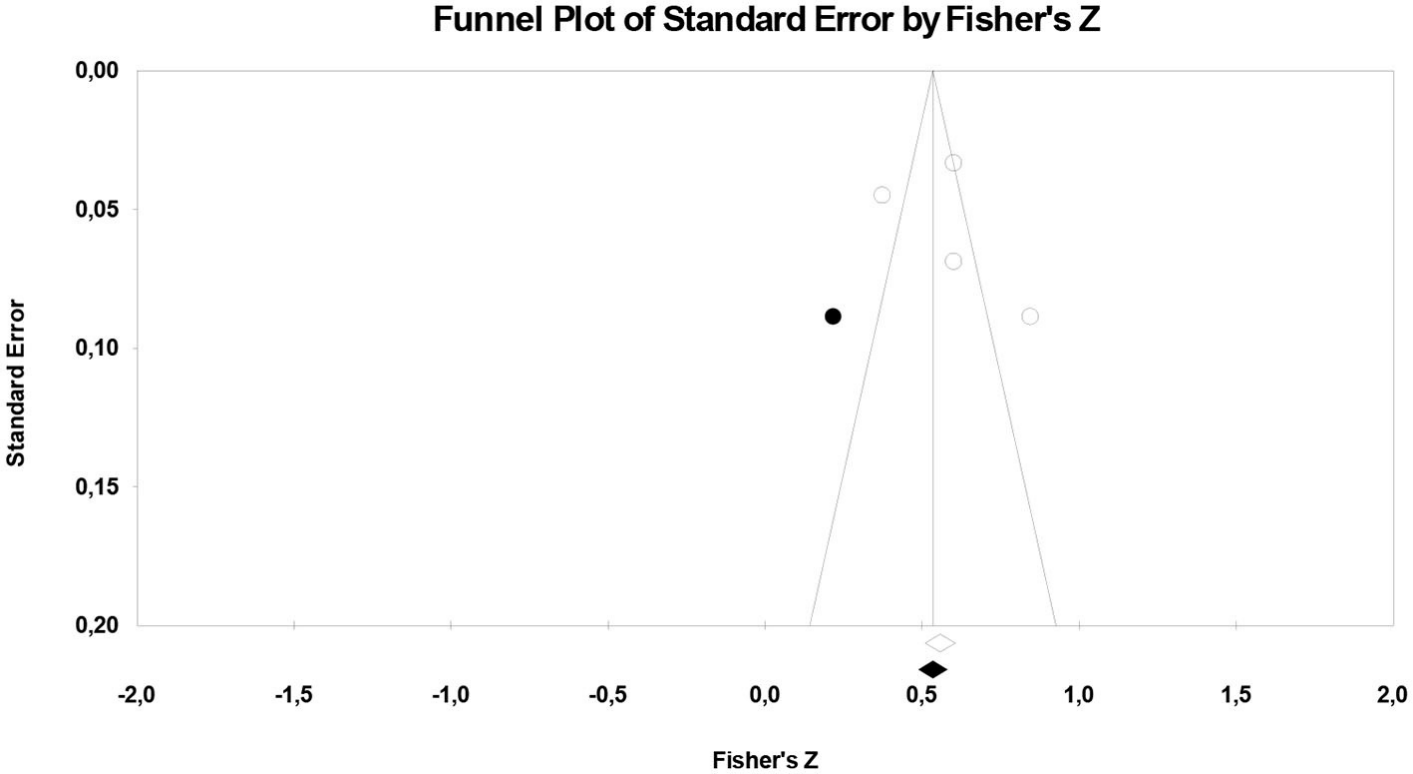
Funnel plot graphic for general irrational beliefs.

In order to determine whether the effect size obtained was appropriate for the purpose, the research data were analyzed with Duval and Tweedie's (2000) trim-and-fill method (Figure 3).

When Figure 3 is considered, it can be said that the funnel plot drawn for the studies included in the study is symmetrical. According to Duval and Tweedie's (2000) trim-and-fill method, the number of hypothetical studies that should be added into the analysis to avoid publication bias is 1. This shows that there is not any publication bias. In addition, Rosenthal's (1979) “fail-safe *N*” number was also calculated to assess publication bias (Rothstein et al., 2005). The fail-safe *N* number was calculated as 219 at 0.05 confidence level. This means that at least 219 studies with contradictory results must be found in the literature to invalidate the results of the present meta-analysis. The number of 219 is more than seven times higher than the value of 30 obtained by the formula 5k + 10 (*k* = 4) (Fragkos et al., 2014). This finding reveals that the results are valid.

#### 3.1.2 The results of the sub-group analysis

In addition to the overall effect size analysis, a subgroup analysis was conducted to determine the source of heterogeneity in the findings. In the subgroup analysis, it was examined whether the country in which the study was conducted made a significant difference. Analog to ANOVA results on whether the country in which the study was conducted affected the relationship between parents' irrational beliefs and general irrational beliefs are reported in Table 4.

**Table 4:** Sub-group analysis results.

**Group**	***k***	**Fisher *z***	***SE***	***p***	**%95 CI**		***df***	***Q_*B*_***	***p***
					**Lower limit**	**Upper limit**			
Romania	1	0.60	0.069	0.000	0.469	0.740	2	7.27	0.03
Turkey	2	0.49	0.114	0.000	0.270	0.715			
The USA	1	0.85	0.089	0.000	0.673	1.023			
Overall	4	0.66	0.002	0.000	0.561	0.754			

The results indicate that there is a statistically significant difference between the subgroups depending on the country of the study [_*Q*_*B*__(2)__ = 7.27, *p* < 0.05]. Among the countries included in the analysis, the highest effect size was 0.85 from the USA and the lowest effect size was 0.49 from Turkey.

### 3.2 A meta-analysis of the relationships between parents' irrational beliefs and parents' negative mental health characteristics

This section presents the results of the meta-analysis of the relationships between six different studies that examine negative mental health characteristics of parents and parental irrational beliefs. Firstly, effect sizes related to the correlations between Parents “Irrational Beliefs and Parents” Negative Mental Health Characteristics were calculated and shown in Table 5.

**Table 5:** The results of the overall effect size about parents' negative mental health characteristics.

**Model**	***k***	**Fisher *z***	***z***	***SE***	***p***	**%95 CI**		***df***	***Q***	***p***	***I* ^2^**
						**Lower limit**	**Upper limit**				
Fixed	6	0.273	10.06	0.027	0.000	0.220	0.326	5	26.42	0.00	81.08
Random	6	0.243	4.97	0.080	0.000	0.242	0.557				

The effect size of the data of the studies included in the meta-analysis was estimated at between 0.22 and 0.33 as 0.27 with a 95% confidence interval, based on the fixed effects model. According to the random effects model, it was estimated at between 0.24 and 0.56 as 0.24 with a 95% confidence interval. The calculated values indicate a medium level effect. The *Q* (*df* = 3) statistic for this analysis was calculated as 26.42 (*p* < 0.01). The estimated *I*^2^ value is 81.08%. The values indicate the heterogeneity of the studies included in the analysis.

**Figure 4:**
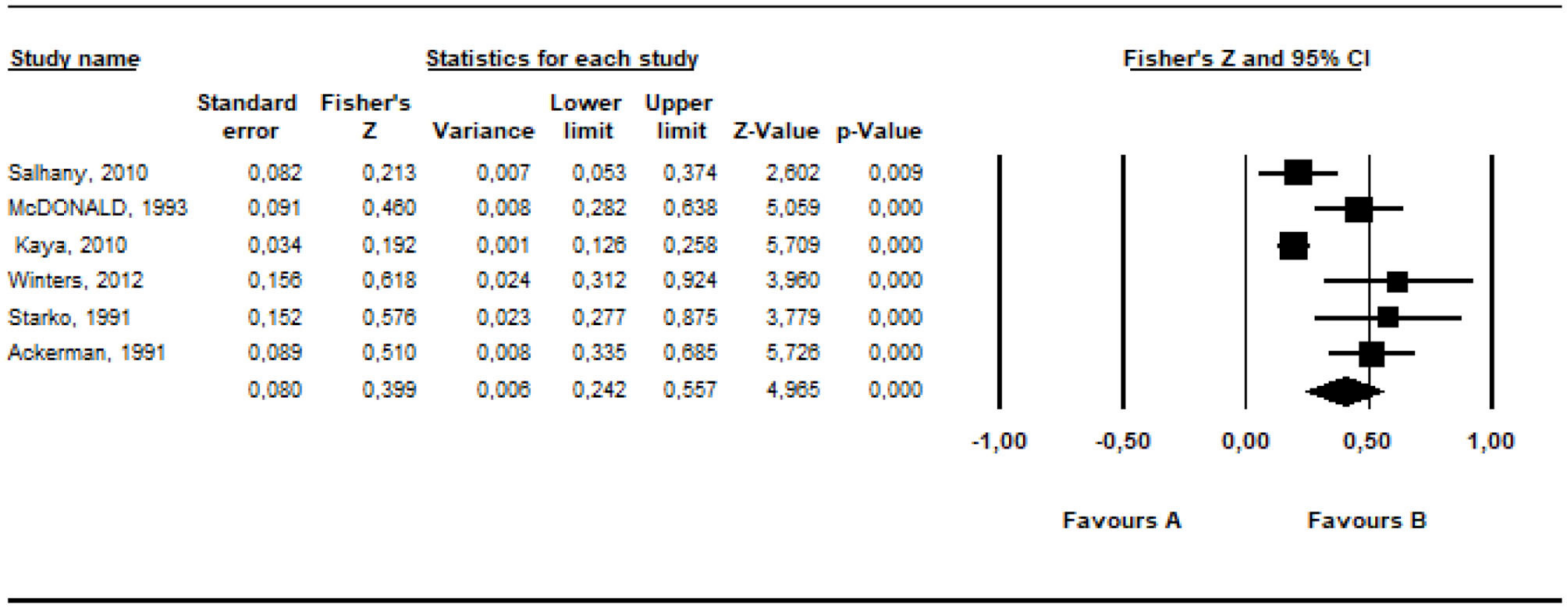
Forest plot showing the distribution of the effect size of the studies.

The *r* equivalent of the estimated Fisher *z* value of 0.178 was calculated as 0.176. This value is considered as a small effect according to Cohen (1988). The forest plot showing the distribution of effect sizes according to the random effects model is presented in Figure 4.

According to the forest graph shown in Figure 4, the largest estimated effect size was calculated as 0.15 by Winters (2012) and the lowest value was calculated as 0.03 by Kaya (2010). In addition, all of the estimated effect sizes are positive.

#### 3.2.1 The results of the validity of effect size estimates

To determine whether the average effect size estimates obtained for the present research are valid, the funnel plot of Duval and Tweedie (2000) was examined by the trim and fill method (Rothstein et al., 2005).

**Figure 5:**
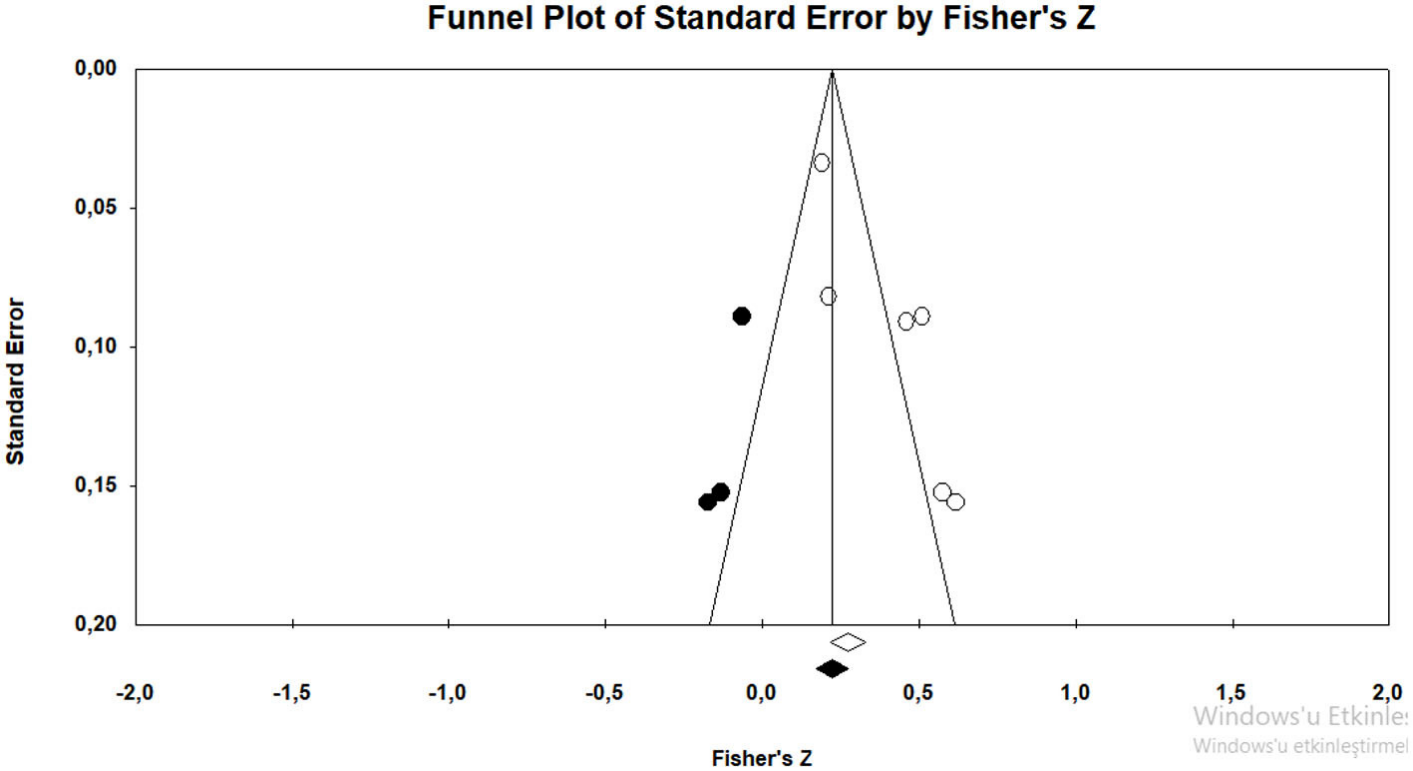
Funnel plot graphic for parents' negative mental health characteristics.

According to the Figure 5, it can be stated that the funnel plot drawn for the studies in the present research is symmetrical. According to Duval and Tweedie's (2000) trim and fill method, there are three hypothetical studies that should be added into the analysis to avoid publication bias. This indicates that there is not any publication bias. In addition, Rosenthal's (1979) “fail safe N” number was also calculated to assess publication bias (Rothstein et al., 2005). The fail-safe N number was calculated as 120 at the 0.05 confidence level. This implies that at least 182 studies with contradictory results must be found in the literature to invalidate the results of the present meta-analysis. The number 182 is three times the value of 40 obtained with the formula 5k + 10 (*k* = 6) (Fragkos et al., 2014). This finding reveals that the results are valid.

#### 3.2.2 The results of the sub-group analysis

In addition to the overall effect size analysis, subgroup analysis was conducted to determine where the heterogeneity of the findings stemmed from. In the subgroup analysis, it was examined whether the country where the study was conducted made a significant difference. The analog to ANOVA results of the country of study on the correlation between parents' irrational beliefs and parents' negative mental health characteristics are presented in Table 6.

**Table 6:** Sub-group analysis.

**Group**	***k***	**Fisher *z***	***SE***	***p***	**%95 CI**		***df***	***Q_*B*_***	***p***
					**Lower limit**	**Upper limit**			
Canada	3	0.221	0.069	0.000	0.410	0.680	2	21.397	0.000
Turkey	1	0.192	0.034	0.000	0.126	0.258			
The USA	2	0.333	0.123	0.007	0.092	0.575			
Overall	6	0.202	0.264	0.000	0.207	0.322			

The results revealed a significant difference between the correlation values obtained depending on the country of the study [_*Q*_*B*__(2)__ = 21.397, *p* < 0.05]. Among the countries included in the analysis, the highest effect size was 0.33 from the USA and the lowest effect size was 0.19 from Turkey.

### 3.3 A meta-analysis of the associations between parents' irrational beliefs and children's negative mental health characteristics

This section presents a meta-analysis of the associations between parental irrational beliefs and some negative mental health characteristics of children, such as depression and anxiety. Initially, the overall effect size of the correlations obtained from four different studies was calculated and the data obtained are shown in Table 7.

**Table 7:** The results of the overall effect size about children's negative mental health characteristics.

**Model**	***k***	**Fisher *z***	***z***	***SE***	***p***	**%95 CI**		***df***	***Q***	***p***	***I* ^2^**
						**Lower limit**	**Upper limit**				
Fixed	4	0.145	5.394	0.027	0.000	0.092	0.197	3	4.966	0.174	39.587
Random	4	0.150	4.122	0.036	0.000	0.079	0.222				

The effect size of the data of the studies included in the meta-analysis was estimated at between 0.09 and 0.20 as 0.15 with a 95% confidence interval, based on the fixed effects model. According to the random effects model, it was estimated at between 0.04 and 0.22 as 0.15 with a 95% confidence interval. The calculated values indicate a low-level effect (Evans, 2023). The common way to assess whether the studies included in the meta-analysis are heterogeneous is the *Q* test (Huedo-Medina et al., 2006). For this analysis, the *Q* (*df* = 3) statistic was estimated as 4.97 (*p* < 0.05). However, for more comprehensive information on heterogeneity, the *I*^2^ value is examined. The estimated *I*^2^ is 39.59%. 60% of the variance between the studies included in the analysis can be explained by random errors. The results show that the studies included in the meta-analysis examine the relationship between parents' irrational beliefs and children's negative mental health characteristics are not heterogeneously distributed. Therefore, subgroup analyses were not conducted for this relationship.

**Figure 6:**
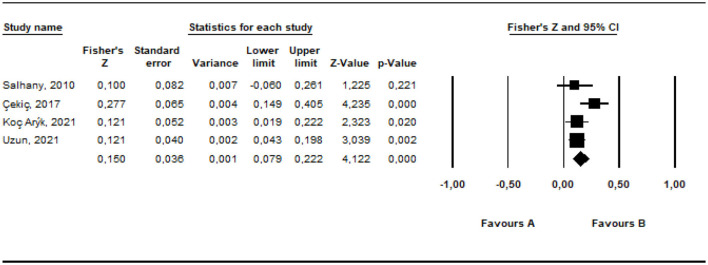
Forest plot graphic for children's negative mental health characteristics.

The *r* equivalent of the calculated Fisher *z* value of 0.150 was calculated as 0.149. This value is defined as small effect (Cohen, 1988). The forest plot showing the distribution of effect sizes according to the random effects model is presented in Figure 6.

According to the forest graph shown in Figure 6, the largest estimated effect size was calculated as 0.28 by Çekiç et al. (2017) and the lowest value was calculated as 0.10 by Salhany (2010). In addition, all of the estimated effect values are positive.

#### 3.3.1 The results of the validity of effect size estimates

**Figure 7:**
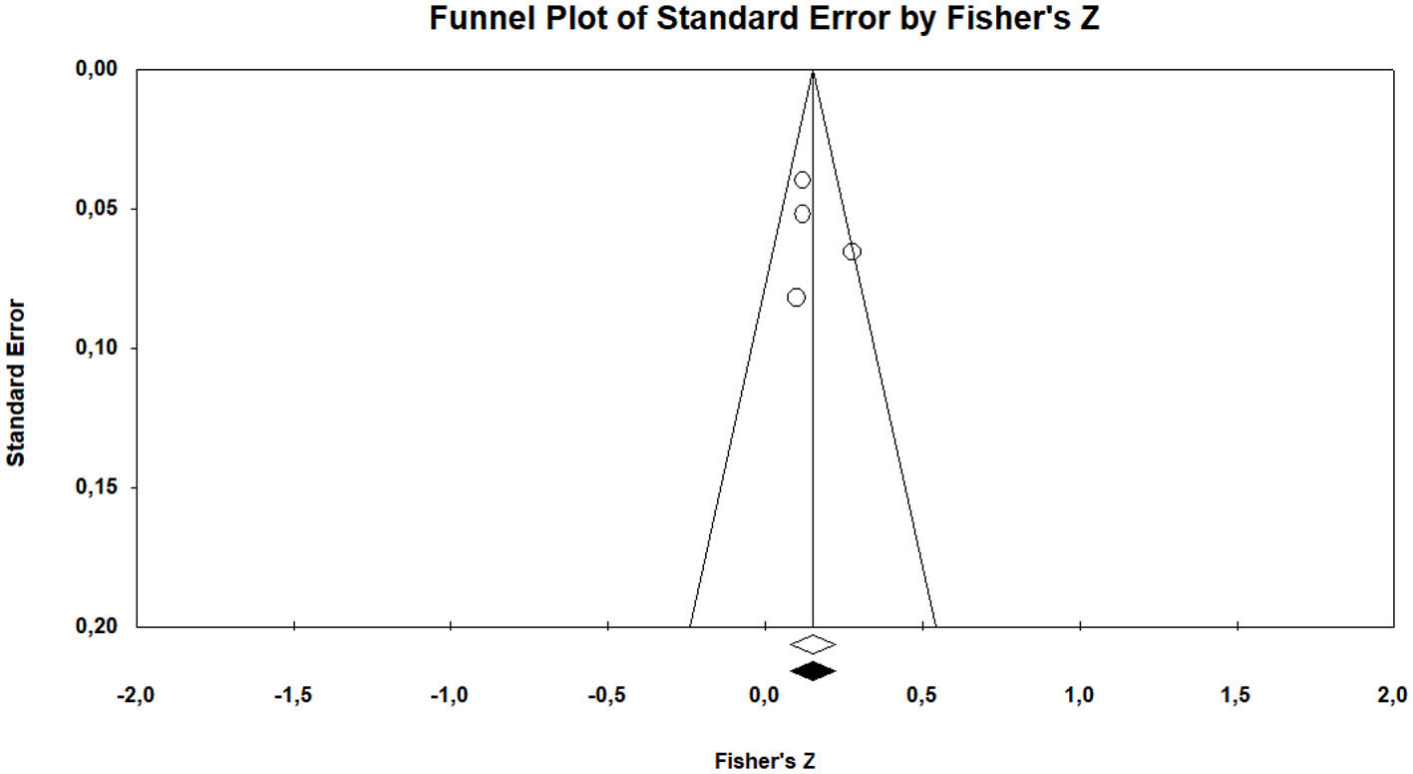
Funnel plot graphic for children's negative mental health characteristics.

In order to determine whether the average effect size estimates from the research are valid or not, Duval and Tweedie's (2000) funnel plot were examined with the trim and fill method (Rothstein et al., 2005). The results are shown in Figure 7.

It can be stated that the funnel plot drawn for the studies in the present study is symmetrical. According to Duval and Tweedie's (2000) trim and fill method, the hypothetical research that is required to be added into the analysis to avoid publication bias is zero. This indicates that there is not any publication bias. In addition, Rosenthal's (1979) “fail-safe N” number was also calculated to assess publication bias (Rothstein et al., 2005). The fail-safe N number was calculated as 27 at the 0.05 confidence level. This implies that at least 27 studies with contradictory results must be found in the literature to invalidate the results of the present meta-analysis. The number 27 is higher than the value of 26 obtained with the formula 4k + 10 (*k* = 4) (Fragkos et al., 2014). This finding reveals that the results are valid. The number 27 is higher than the value of 26 obtained from the formula 4k + 10 (*k* = 4) (Fragkos et al., 2014). This finding also reveals that the results are valid.

It shows that the studies included in the meta-analysis to examine the relationship between parents' irrational beliefs and children's negative mental health characteristics are not heterogeneously distributed. Therefore, subgroup analyses were not conducted for this relationship.

### 3.4 Meta-analysis of the relationship between parents' irrational beliefs and positive parental behaviors

In this section, a meta-analysis on the relationships between parents' irrational beliefs and positive parental behaviors was conducted. First, the overall effect size for the correlation values obtained from five different studies was calculated and presented in Table 8.

**Table 8:** The results of the overall effect size about parents' positive behaviors.

**Model**	***k***	**Fisher *z***	***z***	***SE***	***p***	**%95 CI**		***df***	***Q***	***p***	***I* ^2^**
						**Lower limit**	**Upper limit**				
Fixed	5	−0.098	−3.429	0.028	0.001	−0.153	−0.042	4	19.513	0.001	79.501
Random	5	−0.164	−2.314	0.071	0.021	−0.303	−0.025				

The effect size of the data from the studies included in the meta-analysis was estimated at between −0.153 and −0.042 as −0.098 within the 95% confidence interval, based on the fixed effects model. According to the random effects model, it was estimated at between −0.303 and −0.025 as −0.164 within the 95% confidence interval. The calculated values indicate a low-level effect. The common way to assess whether the studies included in the meta-analysis are heterogeneous is the *Q*-test (Huedo-Medina et al., 2006). For this analysis, the *Q* (*df* = 4) value was estimated as 19.51 (*p* < 0.01). However, for more comprehensive information on heterogeneity, it is required to check the *I*^2^ value. The estimated *I*^2^ is 79.50%. 20% of the variance between the studies included in the analysis can be accounted for random errors. The results revealed that the studies included in the meta-analysis examine the relationship between parental irrational beliefs and parents' positive behavioral characteristics were heterogeneously distributed.

**Figure 8:**
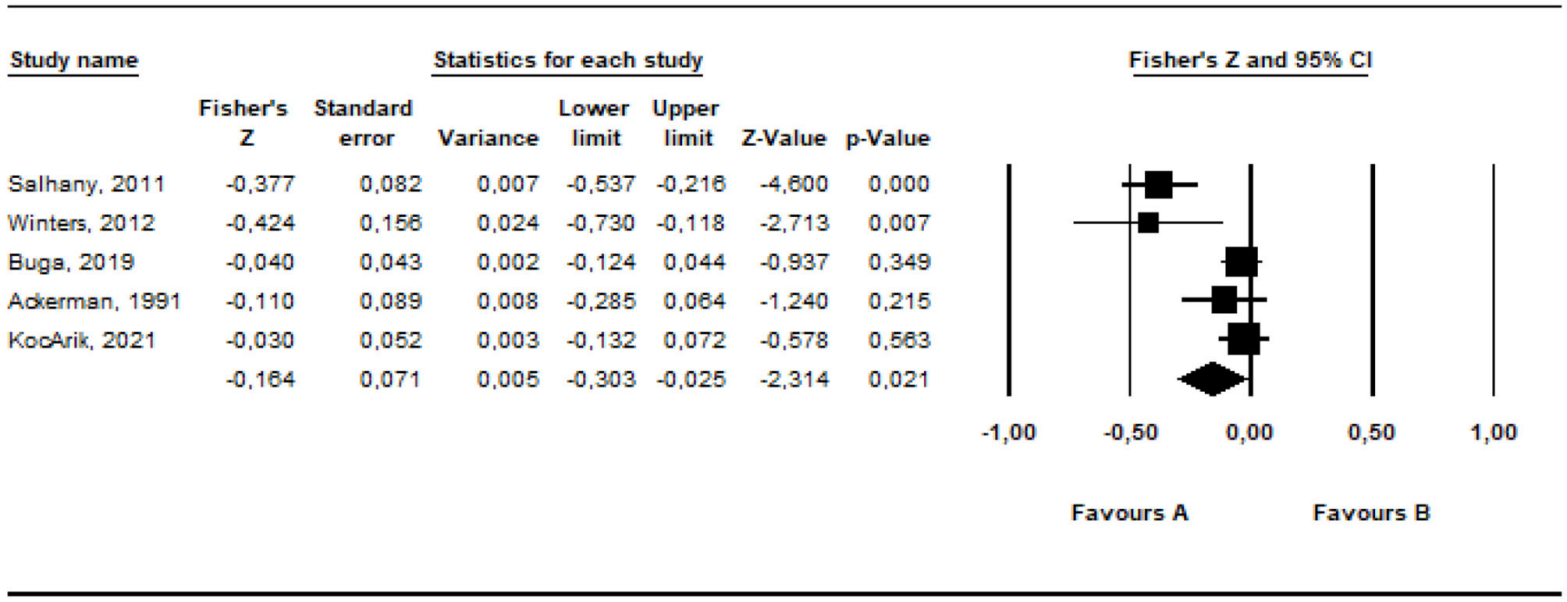
Forrest plot graphic for positive parenting behaviors.

The *r* equivalent of the calculated Fisher *z* value of −0.164 was calculated as −0.163. This value indicates that the relationship between the variables included in the meta-analysis is small. The forest plot showing the distribution of effect sizes according to the random effects model is presented in Figure 8.

According to the forest graph shown in Figure 8, the largest estimated effect size was calculated as −0.424 by Winters (2012) and the lowest value was calculated as −0.040 by Buga et al. (2019). The estimated effect values are negative.

#### 3.4.1 The results of the validity of effect size estimates

In order to determine whether the average effect size estimates obtained as a result of the research are valid, the funnel plot of Duval and Tweedie (2000) was examined by the trim and fill method (Rothstein et al., 2005).

**Figure 9:**
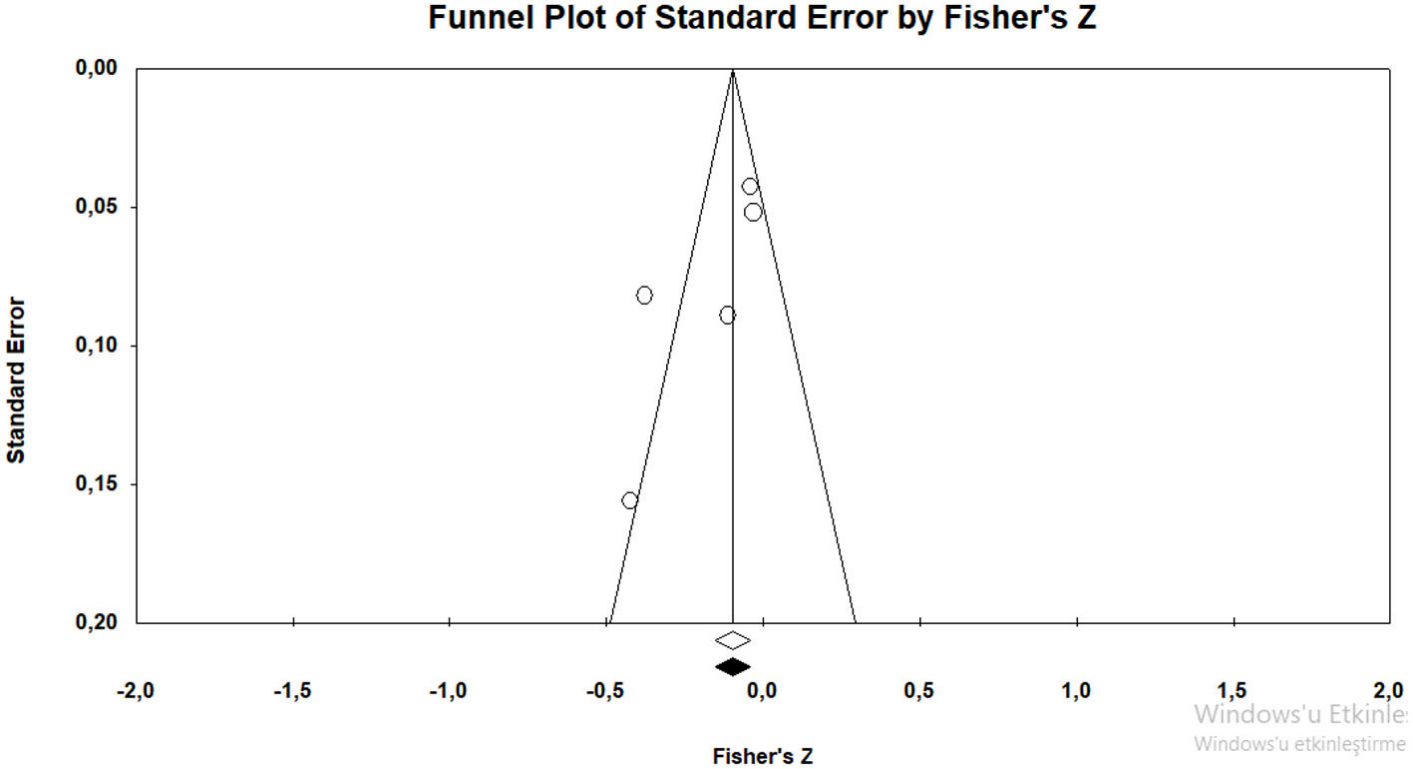
Funnel plot graphic for positive parenting behaviors.

According to the funnel plot graphic shown in Figure 9, it can be stated that the studies included in the research are symmetrical. According to Duval and Tweedie's (2000) trim and fill method, the hypothetical research that is required to be added into the analysis to avoid publication bias is zero. This indicates that there is not any publication bias. Orwin's “fail safe N” number was also calculated to assess publication bias. The number of studies that is required to reduce the calculated effect size to −0.001 trivial value was calculated as 483. This value indicates that at least 483 studies with contradictory results must exist in the literature to invalidate the results of the present meta-analysis. The number 483 is more than 10 times the value of 35 obtained with the formula 5k + 10 (*k* = 5) (Fragkos et al., 2014). The values show that the values obtained by the present meta-analysis are valid.

#### 3.4.2 The results of the sub-group analysis

In order to determine where the heterogeneity of the findings stemmed from, subgroup analysis was conducted in addition to the overall effect size analysis. In the subgroup analysis, we initially examined whether the country in which the study was conducted created a significant difference. The results of the analog to ANOVA on the correlation between parents' irrational beliefs and parents' positive behavioral characteristics depending on the country of study are presented in Table 9.

**Table 9:** Sub-group analysis.

**Group**	***k***	**Fisher *z***	***SE***	***p***	**%95 CI**		***df***	***Q_*B*_***	***p***
					**Lower limit**	**Upper limit**			
Canada	2	−0.241	0.154	0.119	−0.543	0.062	2	15.899	0.000
Turkey	2	−0.036	0.033	0.275	−0.101	0.029			
The USA	1	−0.377	0.082	0.000	−0.537	−0.216			
Overall	5	−0.089	0.030	0.003	−0.148	−0.025			

The results revealed that there was a difference between the subgroups depending on the country of the study [QB(2) = 14.947, *p* < 0.05]. However, the data obtained from Turkey and Canada were not significant. Further studies are required to determine whether the relationships between parents' irrational beliefs and positive parental behaviors differ among subgroups.

### 3.5 Meta-analysis of the relationship between parents' irrational beliefs and negative parental behavior

Firstly, effect sizes related to the correlations between Parents 'Irrational Beliefs and Negative Parental Behaviors were calculated and shown in Table 10.

**Table 10:** The results of the overall effect size about negative parenting behaviors.

**Model**	***n***	**Fisher *z***	***z***	***SE***	***p***	**%95 CI**		***df***	***Q***	***p***	***I* ^2^**
						**Lower limit**	**Upper limit**				
Fixed	5	−0.216	−7.510	0.029	0.000	−0.273	−0.160	4	64.509	0.000	95.79
Random	5	−0.346	−2.690	0.128	0.007	−0.598	−0.094				

The effect size of the data from the studies included in the meta-analysis was estimated at between −0.273 and −0.160 as −0.216 with a 95% confidence interval, based on the fixed effects model. According to the random effects model, it was estimated at between −0.598 and −0.094 as −0.346 within the 95% confidence interval. The estimated effect sizes are moderate [QB(5) = 64.51, *p* < 0.05]. The common way to assess whether the studies included in the meta-analysis are heterogeneous is the *Q*-test (Huedo-Medina et al., 2006). For this analysis, the *Q* (*df* = 4) value was estimated as 64.51 (*p* < 0.01). However, for more comprehensive information on heterogeneity, it is required to check the *I*^2^ value. The estimated *I*^2^ is 95.79%. 5% of the variance between the studies included in the analysis can be accounted for random errors. The results obtained indicate that the studies included in the meta-analysis to examine the relationship between parental irrational beliefs and parents' positive behavioral characteristics are heterogeneously distributed.

**Figure 10:**
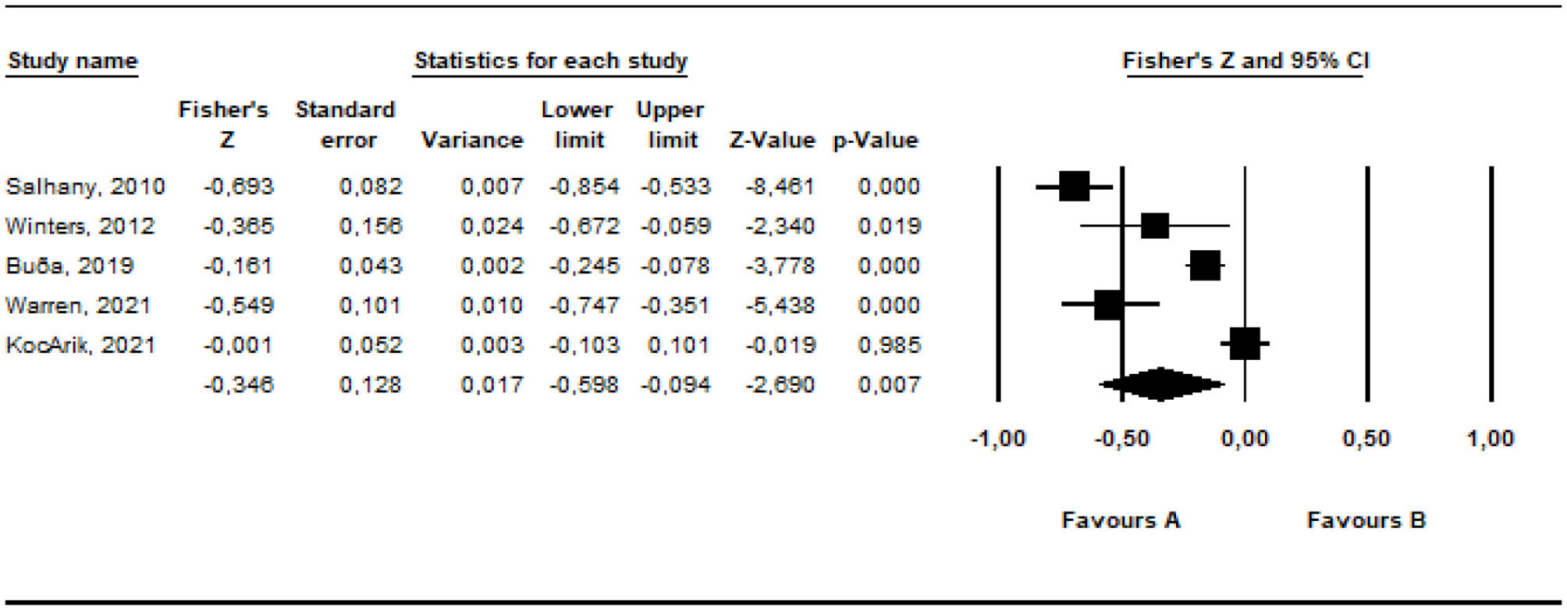
Forrest plot graphic for negative parenting behaviors.

The *r* equivalent of the calculated Fisher *z* value of −0.346 was calculated as −0.333. This value shows that there is a moderate relationship between the variables included in the meta-analysis. The forest plot showing the distribution of effect sizes according to the fixed effects model is presented in Figure 10.

According to the forest graph given in Figure 10, the largest estimated effect size was found as −0.693 by Salhany (2010) and the lowest value was found as −0.161 by Buga et al. (2019). The estimated effect values are negative.

#### 3.5.1 The results of the validity of effect size estimates

**Figure 11:**
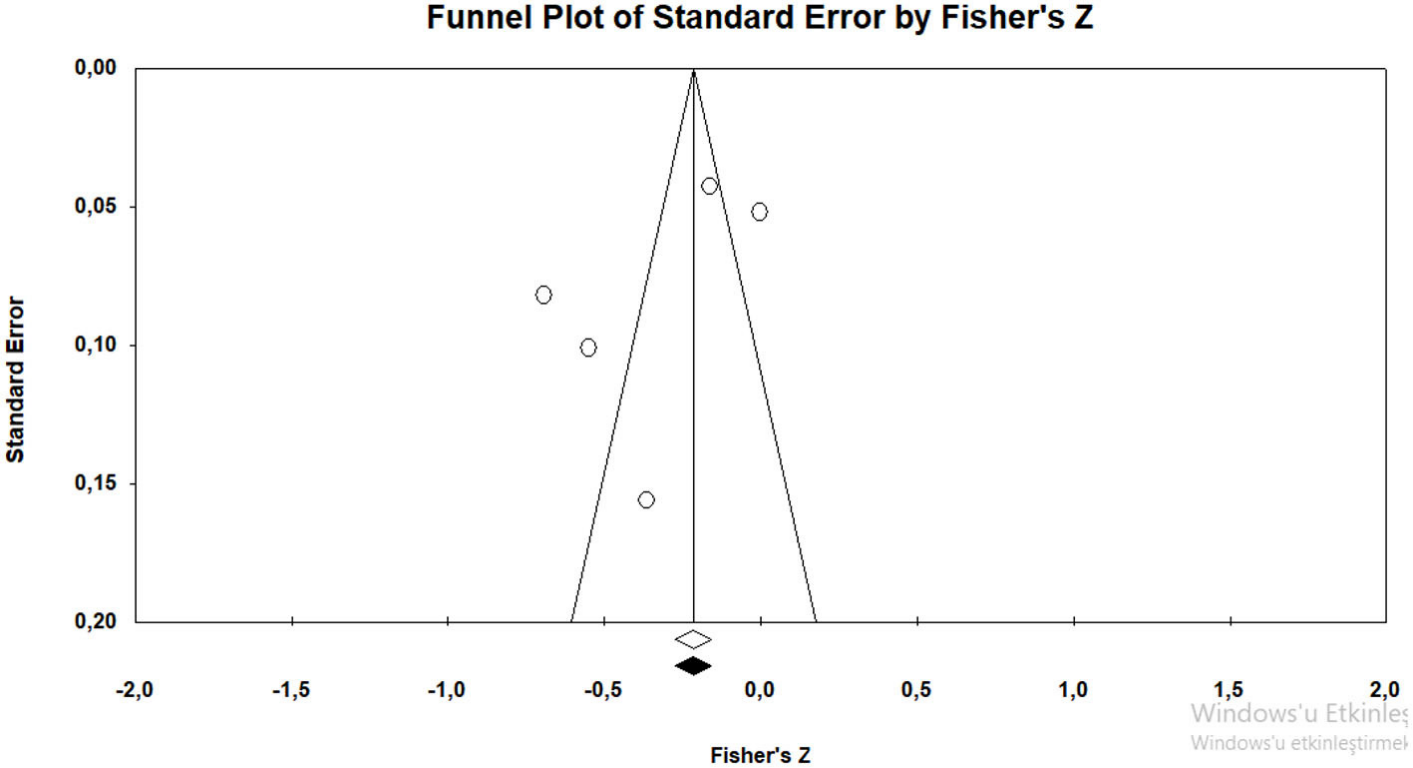
Funnel plot graphic for positive parenting behaviors.

In order to determine whether the average effect size estimates obtained from the research are valid, the funnel plot of Duval and Tweedie (2000) was examined with the trim and fill method (Rothstein et al., 2005) (Figure 11).

It can be stated that the funnel plot drawn for the studies in the present study is symmetrical. According to Duval and Tweedie's (2000) trim and fill method, the hypothetical research that is required to be added into the analysis to avoid publication bias is zero. This indicates that there is not any publication bias. Orwin's “fail-safe N” number was also calculated to assess publication bias. The fail-safe N number was calculated as 100 at the 0.05 confidence level. This value means that at least 100 studies with contradictory results must exist in literature to invalidate the results of this meta-analysis. The number 100 is approximately three times the value of 35 obtained with the formula 5k + 10 (*k* = 5) (Fragkos et al., 2014). The values obtained show that there is not any publication bias in the studies included in the meta-analysis.

#### 3.5.2 The results of the sub-group analysis

In order to determine where the heterogeneity of the findings stemmed from, subgroup analysis was conducted in addition to the overall effect size analysis. In the subgroup analysis, we initially examined whether the country in which the study was conducted made a significant difference. The results of the analog to ANOVA on the correlation between parents' irrational beliefs and parents' negative behavioral characteristics in terms of the country of study are presented in Table 11.

**Table 11:** Sub-group analysis.

**Group**	***k***	**Fisher *z***	***SE***	***p***	**%95 CI**		***df***	***Q_*B*_***	***p***
					**Lower limit**	**Upper limit**			
Canada	1	−0.0365	0.156	0.019	−0.672	−0.059	2	26.396	0.000
Turkey	2	−0.084	0.080	0.295	−0.241	0.073			
The USA	2	−0.633	0.071	0.000	−0.772	−0.494			
Overall	5	−0.389	0.050	0.000	−0.488	−0.291			

The results showed that there is a difference between subgroups depending on the country of the study [_*Q*_*B*__(2)__ = 26.396, *p* < 0.05]. However, the data obtained from Turkey are not significant. Further studies are required to determine whether the associations between parental irrational beliefs and negative parental behaviors differ among subgroups.

## 4 Discussion

In the present study, it was aimed to examine the variables related to parents' irrational beliefs through meta-analysis. In the systematic review, 15 different studies on the relationships of some variables related to parental irrational beliefs were found. In these studies, the relationships of parental irrational beliefs with 23 different variables were investigated. These variables are general irrational beliefs, negative mental health characteristics of parents, some negative mental health characteristics of children such as depression and anxiety, positive parental behaviors and negative parental behaviors. In these 15 studies, the associations between parental irrational beliefs and 23 different variables were examined. These variables are general irrational beliefs (parent), self-acceptance (parent), anxiety (parent), depression (parent), parental stress, positive and negative parental behaviors, couple compatibility level (parent), parent-child relationship, caring for the child, neglectful parenting, life satisfaction (parent), inconsistent discipline, corporal punishment, parental competence, control-oriented parental attitude, psychological violence, adaptive skills (child), problem solving skills (child), irrational beliefs (child), depression (child), anxiety (child), and child's behavioral problems.

According to the evaluations, variables that were thought to measure similar characteristics were grouped. Accordingly, parental irrational beliefs and parental characteristics such as parents' general irrational beliefs, positive and negative mental health characteristics, positive and negative behaviors, child characteristics such as children's irrational beliefs, negative mental health characteristics and positive characteristics, and parent-child relationship and adaptation characteristics that evaluate the process between parent and child were examined. As a result of the analyses, the largest effect size was found in the correlation between parental irrational beliefs and general irrational beliefs (*r* = 0.54), while the smallest effect size was estimated between parental irrational beliefs and positive parental behaviors (*r* = 0.00). The meta-analysis results of the relationship between parental irrational beliefs and negative parental behaviors were not statistically significant (*p* < 0.05). When the effect sizes were compared in terms of the country where the study was conducted, the largest effect size in general irrational beliefs and positive parental behaviors was in the USA, while the lowest effect size was in Turkey. This finding is not a big surprise when the individualistic society like America and the more collectivistic society like Turkey are taken into consideration. The typical behaviors of these two cultures are also defined as independence-interdependence in the related literature. It is inevitable that these characteristics are reflected in mothers' child-rearing behaviors and beliefs about parenting that affect these behaviors (Kagitçibaşi, 2017). When the findings obtained are evaluated from this point of view, the difference in subgroup analyses based on countries regarding irrational beliefs can also be interpreted as the effect of culture.

According to the results of the meta-analysis, the largest effect size was between parental irrational beliefs and general irrational beliefs (*r* = 54, *p* < 0.05). This finding confirms the assumption of Cognitive Behavioral Therapies (Corey, 2005; Sharf, 2011) that irrational, impaired and/or dysfunctional ways of thinking affect the emotions and behaviors of individuals in all aspects. While developing irrational beliefs scales regarding parenting roles, researchers took the beliefs defined by Cognitive Behavioral Therapies as a reference (Kaya and Hamamci, 2011; Gavita, 2011). The correlation values between general irrational beliefs and irrational beliefs developed specifically for a particular situation are measured as medium and high levels in studies (Sari and Korkut Owen, 2015; Gavita, 2011). These results confirm the high effect obtained for the correlation values between parental irrational beliefs and general irrational beliefs.

When the findings of the study are examined, it is revealed that there is a moderate effect between irrational beliefs of parents and negative mental health characteristics of parents. There are also studies on parents' irrational beliefs and parents' negative mental health characteristics in the literature. In the study conducted by Hamamci et al. (2011), a significant difference was found between the ways of coping with stress, self-confidence and helplessness of parents with high and low unrealistic expectations. Starko (1991) reported a statistically significant relationship between parents' irrational beliefs and stress levels. Pochtar (2010) emphasized that parents with more irrational beliefs have higher stress levels. Witt (2005) examined the role of parents' irrational beliefs and autism symptoms in the child's stress level. According to the findings of the study, parents with more irrational beliefs evaluated their children's autism symptoms as more stressful and concluded that these parents had higher overall stress levels. In the study conducted by Çekiç et al. (2019) to explain the relationship between parents' irrational beliefs and life satisfaction, it was revealed that parents' irrational expectations and perfectionist beliefs significantly predicted their life satisfaction. Research generally shows that parents with higher irrational beliefs are more likely to experience stress.

Another finding of the study is the relationship between parents' irrational beliefs and some negative mental health characteristics of children such as depression and anxiety. Studies examining the relationship between parents' irrational beliefs and some negative mental health characteristics of children such as depression and anxiety are found in the literature. Parents' unrealistic beliefs regarding parenting negatively affect their self-efficacy and cause anger. Parents' anger negatively affects their attitudes and behaviors toward their children and causes psychological problems in the children (Kaya and Hamamci, 2011). Isikol (2019) revealed that there is a significant positive relationship between children's problem behaviors and parents' unrealistic beliefs. In the study conducted by Kudu-Arican (2017), it was found that the scores obtained from the perfectionism, emotional harm and expectations subscales of parents who have children with psychological problems were higher than those of parents who have children without psychological problems. In another study, it was found that in their relationships with children, mothers of adolescents with attention deficit hyperactivity disorder had higher level of unrealistic beliefs than the mothers of adolescents without the same disorder (Barkley et al., 1992). In another study, it was found that unrealistic beliefs were higher in mothers of adolescents with substance addiction. According to the study, unrealistic beliefs determine parents' attitudes toward their children and lead their children to have unrealistic expectations (Hojjat et al., 2016).

Another finding of the study was that there was a small effect between parents' irrational beliefs and positive parental behaviors. According to Azar et al. (2008), parents' parenting beliefs significantly affect their parenting roles and their self-perception of their parental role. Bagci (2013) also found a significant negative relationship between parents' irrational beliefs and the dimensions of family functioning such as showing the proper attention and emotional responsiveness. Warren et al. (2018) found a significant positive relationship between parents' irrational beliefs and authoritarian parenting. Pochtar (2010) found that the high level of irrational beliefs was associated with inconsistent parental behaviors.

In the article, subgroup analyses were made according to the country where the study was conducted to determine intercultural differences. According to the results of the analyses on General Irrational Beliefs, the data obtained from all countries where the studies were conducted were significant. In other words, the relationships between irrational beliefs about parenting and general irrational beliefs differ according to the country where the studies were conducted. According to the results, the largest effect size was calculated in the USA with 0.85 and the lowest effect size was calculated in the studies conducted in Turkey. The effect size values calculated for all countries in the relationship between PIB and negative mental health of parents were significant. As in the effect size values calculated for the previous variable, the largest effect size was calculated for the USA, while the effect size value calculated for the studies conducted in Turkey was the lowest. A subgroup analysis by country for the relationships between PIB and children's negative mental health characteristics could not be conducted because the data were not heterogeneous. The effect size values calculated in the subgroup analyses regarding the relationships between PIB and parents' positive behaviors were significant only for the USA, but not for Turkey and Canada. There are two different studies included in the analysis in both countries. In one of these studies, a negative correlation was found between PIB and positive parental behaviors, whereas in the other study a negative correlation was found. A similar situation was found between PIB and negative parental behaviors. In the subgroup analyses of this relationship, while the effect size calculated for the studies conducted in Canada and the USA was significant, the values obtained for Turkey were not significant. More studies need to be included in the meta-analysis for the significance of the data obtained from subgroup analyses regarding the correlation between positive and negative parental behaviors and PIB.

The data obtained from the subgroup analyses reveal that the relationships between PIB and general irrational beliefs and negative mental health characteristics of parents show a significant difference according to the country where the study was conducted. When the effect sizes are taken into consideration, the effect size values calculated for the studies conducted in Turkey from both subgroup analyses are lower. While this situation can be explained by the behaviors of families representing emotional and psychological dependency in the Turkish family structure (Kagitçibaşi, 2017), the individualistic and independent family characteristics of Western cultures such as the USA, Canada or Romania (Markus and Kitayama, 1991) can be considered as the reason for this difference. The fact that the USA, Canada, and Romania are “self-centered” individualistic societies with similar cultural values, while Turkey has a more “other-centered” collectivist culture may change parental cognitions. Çekiç et al. (2021) examined Turkish and Syrian parents' irrational beliefs about parenting behaviors and found that Turkish and Syrian parents gave similar responses to four different scenarios presented to them. This similarity was explained by the common cultural heritage of the two countries. Bornstein et al. (1998) investigated mothers' self-evaluations in child rearing in Argentina, Belgium, France, Israel, Italy, Japan, and the United States and found that parental beliefs are related to cultural background. In this study, it was found that mothers in eastern cultures (e.g., Japan) rated themselves as less competent than mothers in western cultures (e.g., the USA). Furthermore, Japanese mothers were found to be less satisfied with their own parenting compared to mothers in all other countries, despite high levels of investment in their parenting. The cultural background of parental cognitions is exemplified by the humble, self-effacing attitudes of the Japanese and the social unacceptability of making claims about one's own abilities, and by Americans' rewarding individual effort and viewing their own parenting as much a personal achievement as the child's success. This emphasizes the existence of cross-cultural differences in the meaning attributed to parenting. In another study, the parenting cognitions of mothers in two immigrant groups (Japanese immigrants and South American immigrants) living in the USA were examined. While the parenting cognitions of South American immigrant mothers were more similar to the cognitions of mothers in the USA, the cognitions of Japanese immigrant mothers tended to be similar to the cognitions of Japanese mothers or intermediate between Japanese and American mothers (Bornstein and Cote, 2004). Differences in irrational beliefs about parenting among parents living in different countries can also be explained by cultural diversity.

### 4.1 Limitations and suggestions

Our research has some limitations. One of them is that the results obtained are limited to these, since the studies with more significant results were published. The screening of the studies to be included in the meta-analysis can be expanded to include unpublished studies such as theses and research reports.

The effects observed in subgroup analyses between countries may be due not only to cultural differences, but also to the structural characteristics of the measurement tools used or methodological differences. As a matter of fact, some of the sub-dimensions were labeled differently in the measurement tools that were accessed within the scope of this research and that tried to measure irrational beliefs about parenting in different cultures.

This research, which was conducted by including only articles written in Turkish and English, can be repeated by expanding it to include articles written in different languages by different researchers. Especially the inclusion of unpublished studies will increase the level of generalizability of meta-analysis results. In addition, researchers can test the results of meta-analyses obtained in different cultures. For example, the relationship between children's negative mental health characteristics and irrational beliefs about parenting can be examined in different cultures.

## Data Availability

The original contributions presented in the study are included in the article/supplementary material, further inquiries can be directed to the corresponding author.
